# Results from the VIOLIN study: verbal violence against voluntary migrants and refugees in German public institutions, discrimination and their association with mental health—an online-cross-sectional study: Institutional verbal violence and discrimination against migrants and their impact on mental health

**DOI:** 10.1186/s12889-025-24363-y

**Published:** 2025-08-27

**Authors:** Meret Jäschke, Andrea Borho, Eva Morawa, Felicitas Burkhardt, Lucia Romero Gibu, Mojib Atal, Nicolas Rohleder, Silke Jansen, Petra Bendel, Yesim Erim

**Affiliations:** 1 Department of Psychosomatic Medicine and Psychotherapy, University Hospital Erlangen, Friedrich-Alexander-Universität Erlangen-Nürnberg (FAU), Schwabachanlage 6, 91054 Erlangen, Germany; 2 Department of Psychology, Friedrich-Alexander-Universität Erlangen-Nürnberg (FAU), Erlangen, Germany; 3 Department of English, American and Romance Studies, Friedrich-Alexander-Universität Erlangen-Nürnberg (FAU), Erlangen, Germany; 4 Department of Political Science, Friedrich-Alexander-Universität Erlangen-Nürnberg (FAU), Erlangen, Germany

**Keywords:** Verbal violence, Migrants, Refugees, Germany, Discrimination, Mental health, Institution

## Abstract

**Theoretical background:**

Verbal violence and discrimination are psychological stressors for migrants and increase the likelihood of mental illness. This cross-sectional online survey examined the frequency of occurrences of institutional verbal violence (IVV) and discrimination reported by voluntary migrants and refugees in Germany, as well as their association with mental health.

**Methods:**

Adult voluntary migrants and refugees in Germany were recruited for the online survey using snowball and community-based sampling. Measurement instruments included the Everyday Discrimination Scale (EDS), the Patient Health Questionnaire 2 (PHQ-2), the Generalized Anxiety Disorder Scale 2 (GAD-2), and the self-developed 24-item IVV Questionnaire. Voluntary migrants were compared to refugees and women to men. T-tests for independent samples and multiple linear regression analyses were calculated.

**Results:**

The data of 137 refugees and 388 voluntary migrants in Germany were evaluated. Since living in Germany, migrants experienced IVV most frequently in public transport (53%), immigration offices (53%), medical practices (48%), hospitals (41%), and city council/district offices (38%). Female voluntary migrants experienced IVV significantly more frequently than male voluntary migrants (*p* = 0.018). There were no significant differences between refugees and voluntary migrants (*p* = 0.50), nor gender differences among refugees (*p* = 0.69) in their experiences of IVV. Experiences of discrimination were less than once a year per person. Refugees showed significantly more symptoms of clinical depression than voluntary migrants (*p* < 0.001), but the frequency of symptoms of generalized anxiety were comparable in both groups (*p* = 0.08). Being a refugee (β=-0.12; *p* = 0.02), low life satisfaction (β=-0.26; *p* < 0.001), frequent experiences of IVV (β = 0.15; *p* = 0.002) and discrimination (β = 0.23; *p* < 0.001) showed significant association with increased symptoms of depression. Significant predictors for elevated symptoms of generalized anxiety were low life satisfaction (β=-0.24; *p* < 0.001), experiences of IVV (β = 0.24; *p* < 0.001) and discrimination (β = 0.26; *p* < 0.001), as well as a low sense of belonging to the country of origin (β=-0.09; *p* = 0.03).

**Conclusion:**

The results show the need for additional action to reduce IVV against migrants, especially in the identified public institutions focusing on immigration offices, health care institutions and city councils/district offices. Training and supervision could be set up for the employees of these institutions as IVV has a negative impact on mental health.

**Supplementary Information:**

The online version contains supplementary material available at 10.1186/s12889-025-24363-y.

## Introduction

The project Verbal Violence against Migrants in Institutions (VIOLIN) is the first one to examine the impact of institutional verbal violence (IVV) experienced by migrants and refugees in Germany and its interrelationship with their mental health. Verbal violence (VV) can be defined as “linguistic behavior that has detrimental effects on the interlocutor” [1], regardless of whether these effects are intended by the speaker or not. IVV is VV in an institutional context e.g. immigration offices, health care institutions and city councils/district offices. In contrast to this, discrimination refers to the unequal treatment of people on the basis of social or physical characteristics such as origin, skin color, ethnicity, gender, sexual orientation, ethnic or religious practice [2]. It is important to underline that VV and discrimination can co-occur, but they should not be regarded as interchangeable concepts: on the one hand, VV can be directed at an individual without any connection to social or physical characteristics (e.g. between intimate partners); on the other hand, discrimination can manifest through non-verbal means and may not necessarily lead to negative impacts on the individual involved (at least during the encounter). Discrimination acts as a psychological stressor as it is uncontrollable and increases the likelihood of mental illnesses [3, 4]. International research has identified discrimination as the most important factor impairing mental health for migrants after resettlement [5]. There is strong empirical evidence for the link between discrimination and poor mental health [6] in form of development and persistence of depression and anxiety [4, 7–11].

In scientific research in Germany the definition of the Federal Statistical Office of Germany is mostly used, which characterizes people to have a migration background if they or at least one parent was born without German citizenship [12]. Refugees are people who have fled their home country because of persecution for their race, religion, nationality, membership in a particular social group, or on grounds of war and political conflicts [13]. Voluntary migrants, in contrast, leave their country of origin on their own volition [13]. Refugees are more likely to experience depression compared to voluntary migrants [14, 15]. Studies show, that compared to voluntary migrants, refugees are exposed to more pre-migratory stressors such as war and persecution, the flight itself, and post-migratory stressors such as procedures to obtain lawful residency which put a strain on their mental health [3]. In literature female migrants showed significantly higher depression [14, 16] and anxiety symptoms [17] compared to their male counterparts.

Previous research on discrimination against migrants has led to the following results: One in two migrants reports experiences of discrimination [18, 19]. Refugees´ experiences of discrimination are slightly more frequent than among the average migrant population in Germany [20]. Migrants with physical or physiognomic characteristics that tend to be interpreted as indicating a migration background report discrimination in 48% of cases. If certain linguistic features (the so called “accent”) indicate that German is not their first language, 59% report discrimination. Without differences in physiognomy, only 17% report discrimination [21]. Black people name their skin color as the most common reason for discrimination [4]. Country of origin and religious affiliation are also reported to have an influence [4, 18, 21, 22]. In general, migrants most frequently report discriminatory experiences when looking for a job or apprenticeship, as well as during interactions with authorities [19]. More specifically, the highest occurrences of discrimination are perceived in the housing market, followed by interactions with offices/authorities, at the workplace, shops/services and health services [18]. Particularly, in these institutions the person discriminated against is in a dependent role [23].

While there is some research on the impact of discrimination on migrants’ health and wellbeing, the impact of VV, as well as its interaction with discrimination, has not been studied to date. Our study puts special emphasis on institutional contexts, where power hierarchies that facilitate both discrimination and VV are particularly pronounced.

Since migrants in Germany are a heterogeneous group [24], the frequency and perceptions of IVV could differ according to different parameters, e.g. between refugees and voluntary migrants, such as migrant workers. We specifically aimed to examine:

1. The frequence of IVV and discrimination by voluntary migrants and refugees.
2. Mental health of voluntary migrants and refugees.
3. The relation between IVV, discrimination and mental health among voluntary migrants and refugees.
4. Gender specific differences and differences between refugees and migrants regarding the research questions.

## Materials and methods

### Participants

The inclusion criteria for the online survey in Germany were a minimum age of 18 and a migrant background. The Federal Statistical Office of Germany define people to have a migration background if they or at least one parent was born without German citizenship [12]. This includes refugees and voluntary migrants.

### Data collection

VIOLIN is an interdisciplinary project of the Friedrich-Alexander University Erlangen-Nürnberg (FAU), with the participation of researchers from Political science, English, American and Romance Linguistics, Health Psychology and Psychosomatics. Using snowball and community-based sampling, recruitment was done through various channels, such as social media (Facebook, Instagram), clubs, Spanish and French speaking Embassies and Consulates, as well as associations of national groups, churches and public institutions, e.g. integration councils and anti-discrimination bodies. The survey homepage was publicized via internet links and flyers with a QR code. The data collection period was from October 21, 2021 to April 01, 2022. A total of 899 migrants fulfilling the above-mentioned definition of migration background voluntarily participated in the survey.

For this cross-sectional online survey, a questionnaire was developed that contains a main module on IVV and four submodules, covering specific political, linguistic and psychological issues, which are not subject of the present study. The self-developed IVV Questionnaire is part of the main module, which was developed by the English, American and Romance Linguistics team, based on the VIOLIN corpus. At the time when the questionnaire was designed, this was a collection of 52 semi-structured interviews with Spanish speaking migrants on critical communication incidents. The interviews where audio-typed, transcribed and coded according to the Grounded Theory methodology, in order to develop items that were empirically based on the factual experience by different migrant groups in Germany. All items were translated by native speakers into the following nine languages: German, English, Spanish, French, Arabic, Polish, Farsi, Pashto and Turkish. A first translation was carried out by a native speaker of the target language; then, the questionnaire was retranslated into German by a native German speaker. If the retranslation to German was identical or directly equivalent to the original, it was retained. If there were substantial differences, the two translators discussed them to develop a common version. The validation of the IVV Questionnaire is the subject of another research paper. The survey was presented using the academic online survey tool Unipark (www.unipark.com; Tivian, Germany) and took on average 30 min to complete.

### Ethics approval and consent to participate

The study was conducted according to the Declaration of Helsinki principles and approval was obtained from the Ethics Committee of the Medical Faculty of the Friedrich-Alexander University Erlangen-Nürnberg (FAU) (project identification code: 451_19B).

Before participating in the study, informed online consent was obtained from each participant. The consent form outlined that data would be published anonymously.

### Measurement instruments

The following sociodemographic and migration-specific variables were collected: sex (female, male, diverse), age (in years), educational degree (left school without graduating, compulsory school, finished with a diploma, finished secondary school with a diploma), current occupation (German course, integration course, work/occupation, school, study, vocational training/apprenticeship, internship, housewife/househusband, (early) retirement, job search, other, none of the above), German language skills according to the European Reference Frame for Languages (no knowledge, A1, A2, B1, B2, C1, C2, first language), residence status (permanent, temporary, no title, other, not specified), duration of stay (in years) and reason to migrate (flight/forced migration, work, education/further education, study, family and partnership reasons, economic reasons, other). The remaining parts of the survey focused on IVV, experiences of discrimination and mental health.

#### Verbal violence in German public institutions

A qualitative study with Latin American migrants [1], focus groups with pupils with a migration background [25] and interviews of refugees from Afghanistan served as preliminary studies. Through an expert consensus group the most relevant forms of VV as well as the institutions where IVV was experienced most frequently were extracted from the VIOLIN corpus. Finally, 24 items were selected for the IVV, which aimed to determine both the type and frequency of IVV as experienced by migrants in Germany. The items consisted of standardized examples of uncomfortable situations that emerged from the data as representative experiences shared by the participants. For example, “The person ignored me when I spoke to him“, “The person insisted that I speak only German”, “The person spoke to me particularly fast, so I could not understand him/her well”, “The person did not look at me when I spoke“, “The person was making fun of me”, or “The person refused to listen to me”. Frequency was measured using a 5-point Likert scale, ranging from “never” (0) to “very often” (4). The impact of the experience was measured on a 5-point Likert scale, ranging from “not at all” (0) to “very much” (5). For the analysis, the average sum scores were computed. The internal consistency measured by Cronbach’s alpha was very high (0.94).

In addition, it was determined in which public institutions IVV had been experienced. For this purpose, 14 examples were given that had been frequently mentioned in the interviews, i.e. public transport, doctors’ offices, immigration offices, police, school. Participants could choose as many institutions as they wanted and could name additional institutions where they had experienced IVV.

#### Perceived discrimination

Perceived discrimination was assessed using the Everyday Discrimination Scale (EDS). It is a questionnaire that can capture both temporary or long-term occurrences of discrimination experienced in daily life. It can capture even relatively low levels of discrimination [26]. Participants were asked to report how often they had been treated unfairly in their everyday life in the past 12 months using a 6-point Likert scale from “never” (0) to “almost daily” (5) for each out of the 9 items. The following item was added: “Are you treated worse than others in public agencies?”. Higher scores indicated a higher level of perceived discrimination. The internal consistency measured by Cronbach’s alpha was very high (0.92).

#### Depression and generalized anxiety disorder: PHQ-4 (Patient Health Questionnaire - 4)

To assess depression and generalized anxiety symptoms, PHQ-4 (Patient Health Questionnaire - 4) [27], as an abbreviated form of the Patient Health Questionnaire (PHQ-D), was utilized. PHQ-4 can be subdivided into a module for depressive symptoms (PHQ-2) and a module for generalized anxiety symptoms (GAD-2).

The PHQ-2 [28] questioned the frequency of depressed mood and anhedonia in the past two weeks. On a 4-point Likert scale from “not at all” (0) to “almost every day” (3) participants indicated the frequency of experiencing “little interest or pleasure in things” and felt “dejection, melancholy or hopelessness “. Clinically relevant depressive symptoms were defined as a sum score greater than or equal to 3.

The Generalized Anxiety Disorder Scale 2 (GAD-2) [29] as a screening instrument for clinically relevant symptoms of generalized anxiety disorder asks about the frequency of feelings of “nervousness, anxiety, or tension” and “not being able to stop or control one’s worry” during the past 2 weeks on a 4-point Likert scale ranging from “not at all” (0) to “present almost daily” (3) with a cut-off score greater than or equal to 3 of the sum score indicating clinically relevant symptom severity.

For the final sample of 525 participants, the correlation coefficient was 0.68 for PHQ-2 (*p* < 0.001) and 0.74 for GAD-2 (*p* < 0.001).

#### Life satisfaction in Germany

Life satisfaction for the participants’ lives in Germany was measured by a visual analogue scale consisting of following manifestations “not satisfied at all” (0) to “very satisfied” (10).

### Statistical analysis

IBM SPSS Statistic 29 (IBM Corporation, Armonk, NY, USA) was applied for the statistical analysis. Means, standard deviations, ranges and frequencies were calculated for the descriptive analyses. Participants who did not complete the relevant questionnaires were excluded from the sample. The sample of 899 participants was reduced to 528 due to missing answers for items relevant to the research question. Additionally, three participants were excluded due to implausible answers. The investigated sample of 525 migrants in total was again divided into two subgroups, 137 refugees and 388 voluntary migrants, depending on the reason for migration and asylum application.

Based on the available cut-off values, the prevalence rates for assessing clinically relevant symptoms of depression and generalized anxiety were calculated for each questionnaire.

For the analysis of differences between refugees and voluntary migrants and between female and male migrants in terms of their mental health (PHQ-2 and GAD-2), experiences of IVV, experiences of discrimination (EDS) and life satisfaction, we conducted T-tests for independent samples after checking the normal distribution of the examined characteristic. The effect size was calculated using Cohen‘s d (d ≥ 0.2 = small, d ≥ 0.5 = medium and d ≥ 0.8 = large effect size).

Multiple linear regression analyses were calculated, in which the results point to a significant association of IVV experience and discrimination experiences with depressive and generalized anxiety disorder symptoms. Due to multicollinearity with a correlation coefficient of 0.75 between experiencing discrimination and IVV, we conducted two separate regression analyses (Additional file 2 and Table 2) for each outcome variable (PHQ-2 and GAD-2). The significance level was set at *p* ≤ 0.05 in all analyses. For the regression analysis, we excluded three participants who had indicated “diverse” as their gender due to the small size of the subsample.

## Results

### Sample characteristics (Socio-demographic and migration-specific variables)

The sample consisted of refugees (*N* = 137) and voluntary migrants (*N* = 388) from 60 different countries. Among the voluntary migrants, 327 were first-generation and 61 were second-generation migrants. There was a higher proportion of women among the sample of voluntary migrants (70%) than among the refugee sample (32%). The average age of the total sample was 38.2 years (SD = 11.6; range 18–78). 108 refugees (79%) and 353 (91%) voluntary migrants had completed secondary school with a degree. 75% of the sample had a high (at least level B2) German language proficiency. The three most frequently mentioned reasons for migration were family/partnership (30%), flight/forced migration (28%) and education (24%). On average, refugees had lived in Germany for 9.4 years (SD = 10.1) and voluntary migrants for 15.1 years (SD = 13.3).

Further sociodemographic and migrant-specific variables are listed in Table 1.

**Table 1:** Socio-demographic and migration-specific characteristics of the total sample

Variables		Total (*N* = 525; 100%)	Refugees (*N* = 137; 26%)	Voluntary migrants (*N* = 388; 74%)	Women (*N* = 316; 60%)	Men (*N* = 206; 39%)
Sex	Female Male Diverse	316 (60%) 206 (39%) 3 (1%)	44 (32%) 92 (67% 1 (1%)	272 (70%) 114 (29%) 2 (1%)	316 (100%)	206 (100%)
Age in years	Range 18–78	M = 38.2 (SD = 11.6)	M = 36.0 (SD = 11.9)	M = 39.0 (SD = 11.5)	M = 37.9 (SD = 11.0)	M = 38.9 (SD = 12.6)
Marital status	Single Permanent partnership Married Divorced Widowed	139 (27%) 74 (14%) 275 (52%) 34 (6%) 3 (1%)	53 (39%) 8 (6%) 70 (51%) 6 (4%) 0 (0%)	86 (22%) 66 (17%) 205 (53%) 28 (7%) 3 (1%)	77 (24.5%) 54 (17%) 157 (50%) 25 (8%) 3 (1%)	62 (30%) 18 (9%) 117 (57%) 9 (4.5%) 0 (0%)
Educational degree	Left school without degree Compulsory school finished with a diploma Finished secondary school with a diploma	12 (2%) 52 (10%) 461 (88%)	8 (6%) 21 (15%) 108 (79%)	4 (1%) 31 (8%) 353 (91%)	4 (1%) 24 (8%) 288 (91%)	7 (3%) 28 (14%) 171 (83%)
Vocational training	Completed Started, but not yet completed Started, but canceled No	234 (45%) 21 (4%) 5 (1%) 265 (50%)	53 (39%) 9 (7%) 3 (2%) 72 (52%)	181 (46%) 12 (3%) 2 (1%) 193 (50%)	133 (42%) 11 (3.5%) 2 (0.5%) 170 (54%)	100 (48.5%) 10 (5%) 2 (1%) 94 (46%)
Age at immigration in years (*N* = 464; 136 R^1^, 328 VM^2^, 183 W^3^, 183 M^4^)		M = 25.1 (SD = 9.1)	M = 26.5 (SD = 10.1)	M = 24.6 (SD = 8.7)	M = 24.8 (SD = 9.3)	M = 25.6 (SD = 8.9)
Duration of stay in years (*N* = 463; 136 R^1^, 327 VM^2^, 183 W^3^, 182 M^4^)		M = 13.4 (SD = 12.7)	M = 9.4 (SD = 10.1)	M = 15.1 (SD = 13.3)	M = 13.9 (SD = 12.4)	M = 13.0 (SD = 13.2)
Five most common countries of origin (*N* = 523; 137 R^1^, 386 VM^2^, 312 W^3^, 205 M^4^)		61 (12%) Germany 53 (10%) Afghanistan 53 (10%) Poland 48 (9%) Syria 41 (8%) Mexico	45 (33%) Syria 44 (32%) Afghanistan 14 (10%) Iran 6 (4%) Poland 5 (4%) Turkey	61 (15%) Germany 47 (12%) Poland 41 (11%) Mexico 25 (6%) Ecuador 24 (6%) Turkey	39 (12.5%) Germany 36 (11.5%) Mexico 29 (9%) Poland 19 (6%) Iran 17 (5.5%) Ecuador	45 (22%) Afghanistan 32 (15.5%) Syria 24 (12%) Poland 22 (11%) Germany 14 (7%) Turkey
German citizenship by birth		46 (9%)	4 (3%)	42 (11%)	27 (8.5%)	19 (9%)
Asylum application (*N* = 382; 117 R^1^, 265 VM^2^, 225 W^3^, 155 M^4^)		109 (28%)	109 (93%)	0 (0%)	31 (14%)	77 (50%)
Residence status (*N* = 382; 117 R^1^, 265 VM^2^, 225 W^3^, 155 M^4^)	Permanent Temporary No title Other Not specified	207 (54%) 126 (33%) 14 (4%) 10 (3%) 25 (6%)	33 (28%) 61 (52%) 13 (11%) 5 (4.5%) 5 (4.5%)	174 (65%) 65 (24.5%) 1 (0.5%) 5 (2%) 20 (8%)	143 (64%) 62 (28%) 2 (1%) 2 (1%) 16 (7%)	64 (41%) 63 (41%) 12 (8%) 8 (5%) 8 (5%)
Reason to migrate (*N* = 465; 137 R^1^, 328 VM^2^, 278 W^3^, 184 M^4^)	Flight/Forced Migration Work Education/Further education Study Family and partnership reasons Economic reasons Other	129 (28%) 72 (15%) 35 (7%) 111 (24%) 139 (30%) 48 (10%) 33 (7%)	127 (93%) 4 (3%) 4 (3%) 7 (5%) 4 (3%) 7 (5%) 11 (8%)	0 (0%) 68 (21%) 31 (9%) 104 (32%) 135 (41%) 41 (12%) 22 (7%)	43 (15.5%) 46 (16.5%) 23 (8%) 69 (25%) 113 (41%) 30 (11%) 12 (4%)	86 (47%) 26 (14%) 11 (6%) 42 (23%) 25 (14%) 17 (9%) 15 (8%)
German language skills (*N* = 461; 130 R^1^, 331 VM^2^, 274 W^3^, 184 M^4^)	No knowledge A1 A2 B1 B2 C1 C2 First language	4 (1%) 24 (5%) 29 (6%) 59 (13%) 95 (21%) 119 (26%) 108 (23%) 23 (5%)	1 (1%) 11 (8%) 9 (7%) 22 (17%) 38 (29%) 32 (25%) 14 (11%) 3 (2%)	3 (1%) 13 (4%) 29 (6%) 37 (11%) 57 (17%) 87 (26.5%) 94 (28.5%) 20 (6%)	2 (1%) 14 (5%) 18 (6.5%) 33 (12%) 48 (17.5%) 75 (27.5%) 70 (25.5%) 14 (5%)	2 (1%) 9 (5%) 11 (6%) 25 (13.5%) 46 (25%) 44 (24%) 38 (21%) 9 (5%)
Recognition of the degree	Complete Partial None Not applicable	198 (38%) 96 (18%) 96 (18%) 135 (26%)	36 (26%) 32 (23%) 34 (25%) 35 (26%)	162 (42%) 64 (16%) 62 (16%) 100 (26%)	123 (39%) 61 (19%) 62 (20%) 70 (22%)	74 (36%) 35 (17%) 33 (16%) 64 (31%)
Occupation	German course Integration course Work/occupation School Study Vocational training/apprenticeship Internship Housewife/househusband (Early) retirement Job search Other None of the above	35 (7%) 12 (2%) 339 (65%) 8 (1%) 88 (17%) 30 (6%) 15 (3%) 41 (8%) 13 (2%) 38 (7%) 11 (2%) 19 (4%)	22 (16%) 8 (6%) 58 (42%) 5 (3.5%) 24 (17.5%) 14 (10%) 9 (6.5%) 6 (4.5%) 5 (3.5%) 12 (9%) 5 (3.5%) 5 (3.5%)	13 (3.5%) 4 (1%) 281 (66%) 3 (1%) 64 (16%) 16 (3%) 6 (1.5%) 35 (9%) 8 (2%) 26 (7%) 38 (10%) 14 (3.5%)	12 (4%) 7 (2%) 214 (68%) 6 (2%) 56 (18%) 14 (4.5%) 8 (2.5%) 36 (11.5%) 2 (0.5%) 22 (7%) 6 (2%) 7 (2%)	23 (11%) 5 (2.5%) 124 (60%) 2 (1%) 32 (15.5%) 16 (8%) 7 (3.5%) 5 (2.5%) 10 (5%) 15 (7%) 2 (1%) 11 (5%)
Form of employment (*N* = 310; 54 R^1^, 256 VM^2^, 195 W^3^, 115 M^4^)	Full-time employment Part-time employment Marginal employment Occasional or irregular employment	217 (70%) 76 (24%) 12 (4%) 5 (2%)	42 (78%) 10 (18%) 2 (4%) 0 (0%)	175 (68%) 66 (26%) 12 (5%) 3 (1%)	116 (59.5%) 65 (33.5%) 11 (5.5%) 3 (1.5%)	101 (88%) 11 (10%) 1 (1%) 2 (2%)
Sense of belonging to country of origin from 0–3		M = 1.99 (SD = 0.91)	M = 1.86 (SD = 0.96)	M = 2.04 (SD = 0.88)	M = 2.07 (SD = 0.86)	M = 1.89 (SD = 0.95)
Sense of belonging to Germany from 0–3		M = 1.74 (SD = 0.85)	M = 1.76 (SD = 0.88)	M = 1.73 (SD = 0.85)	M = 1.69 (SD = 0.88)	M = 1.83 (SD = 0.81)
Future in Germany	Yes No Partly	260 (49%) 67 (13%) 198 (38%)	90 (65.5%) 9 (6.5%) 38 (28%)	170 (44%) 58 (15%) 160 (41%)	139 (44%) 46 (14.5%) 131 (41.5%)	120 (58%) 21 (10%) 65 (31.5%)
Life satisfaction from 0–10		M = 7.1 (SD = 1.94)	M = 6.7 (SD = 2.05)	M = 7.2 (SD = 1.89)	M = 7.18 (SD = 1.89)	M = 7.91 (SD = 2.01)

^1^R: refugees; ^2^VM: voluntary migrants; ^3^W: women; ^4^M: men

#### Verbal violence in German public institutions

The average sum score of the total sample for the 24 items was 25.15 (SD = 18.09). There was no significant difference (t(524) = 0.67; *p* = 0.50; d = 0.07) between refugees (M = 26.04; SD = 18.46) and voluntary migrants (M = 24.84; SD = 17.97). However, female voluntary migrants experienced significantly more IVV (M = 26.42; SD = 18.02) than male voluntary migrants (M = 20.51; SD = 16.56; t(385) = 3.01; *p* = 0.003; d = 0.34). There were no significant differences in the frequency of IVV experienced by female (M = 26.70; SD = 16.16) and male (M = 25.38; SD = 19.34) refugees (t(135) = 0.39; *p* = 0.69; d = 0.07).

IVV was most experienced in the following settings: public transport (53%), immigration offices (53%), doctors’ offices (48%), hospitals (41%), and city council/district offices (38%). No significant differences were found in frequency, occurrence and location of IVV between any of the different sub-groups (voluntary migrants and refugees, women and men).

#### Discrimination

The participants reported perceiving discrimination (EDS) in Germany less than once a year (M = 11.97; SD = 9.63) without a significant difference (t(524) = 0.97; *p* = 0.33; d = 0.10) between refugees (M = 12.66; SD = 10.27) and voluntary migrants (M = 11.73; SD = 9.40). Gender differences were not significant (t(135) = −0.48; *p* = 0.63; d = −0.09) for female (M = 11.95; SD = 8.62) and male (M = 12.86; SD = 10.98) refugees. There were also no significant differences in the experience of everyday discrimination (t(385) = 1.16, *p* = 0.25; d = 0.13) for female (M = 12.03; SD = 9.15) and male voluntary migrants (M = 10.82; SD = 9.90).

#### Depression

One hundred and sixty-five of the 525 participants (31%) showed clinically relevant depressive symptoms (PHQ-2 ≥ 3), 42% of refugees and 28% of voluntary migrants. The average value for depressive symptoms was 2.09 (SD = 1.70). Refugees (M = 2.53; SD = 1.72) presented significantly more depressive symptoms than voluntary migrants (M = 1.94; SD 1.66; t(524) = 3.51; p **<** 0.001; d = 0.35). The results revealed no significant differences in the prevalence of depressive symptoms between female (M = 2.36; SD = 1.56) and male (M = 2.60; SD = 1.80; t(135) = −0.74; *p* = 0.46; d = −0.14) refugees. There were no significant differences regarding depressive symptoms between female (M = 1.96; SD = 1.67) and male (M = 1.87; SD = 1.64; t(385) = 0.47; *p* = 0.64; d = 0.05) voluntary migrants. Among the nine most common countries of origin, Syria (M = 2.63; SD = 1.79), followed by Iran (M = 2.19; SD = 1.90), revealed the highest mean for depressive symptoms. In an additional file [see Additional file 1] further mean values for the nine most common countries of origin in the study sample are provided.

#### Generalized anxiety symptoms

One hundred and fifty-four of 525 participants (29%) in the total sample had clinically relevant anxiety (GAD-2 ≥ 3). This corresponded to 34% of refugees and 28% of other migrants. The mean score of the total sample was 2.05 (SD 1.76) on the GAD-2 scale. The T-test indicated no significant differences between refugees (M = 2.28; SD = 1.77) and voluntary migrants (M = 1.97; SD = 1.76; t(524) = 1.75; *p* = 0.08; d = 0.17) or between female (M = 2.11; SD = 1.77) and male (M = 2.38; SD = 1.77; t(135) = −0.82; *p* = 0.41; d = −0.15) refugees or female (M = 2.04; SD = 1.76) and male (M = 1.78; SD = 1.73; t(385) = 1.31; *p* = 0.19; d = 0.15) voluntary migrants. However, when looking at the mean scores for the individual countries of origin, differences are detectable. Migrants from countries such as Syria (M = 2.54; SD = 1.92), Iran (M = 2.19; SD = 0.67) or Afghanistan (M = 2.10; SD = 1.76) demonstrated the highest levels of anxiety disorder symptoms in our sample. Mean scores for the nine most common countries of origin can be found in an additional file [see Additional file 1].

### Predictors of depression and generalized anxiety symptoms

We conducted multiple regression analyses to examine the association between IVV and mental health. Sociodemographic and migration-specific variables such as migrant group (refugee or voluntary migrant), experience of IVV, gender, age, marital status, educational degree, occupation, knowledge of German, sense of belonging to the country of origin, sense of belonging to Germany, and life satisfaction were considered. The results are presented in Table 2.

Because of the high correlation between IVV and perceived discrimination, separate multiple regression analyses were conducted. Their objective was to examine the relationship between perceived discrimination and psychological well-being. The results can be found in an additional file [see Additional file 2] as the results are similar to those of the regression analysis with IVV as a predictor variable.

**Table 2:** Multiple linear regression analyses including IVV and socio-demographic and migration-related characteristics as predictor variables

Predictors of Depressive Symptoms^1^	B^3^	SE^4^	β	*P*	95% CI^5^
Institutional verbal violence^6^	0.014	0.004	0.152	**0.002**	0.01 to 0.02
Migrant group^7^	− 0.442	0.184	− 0.116	**0.017**	− 0.80 to − 0.08
Life satisfaction^8^	− 0.228	0.046	− 0.263	**< 0.001**	− 0.32 to − 0.14
Sense of belonging to the country of origin^9^	− 0.129	0.084	− 0.067	0.123	− 0.29 to − 0.04
Sex^10^	0.111	0.162	0.032	0.493	− 0.21 to 0.43
Age	− 0.005	0.008	− 0.032	0.514	− 0.02 to 0.01
Marital status^11^	0.029	0.165	0.008	0.861	− 0.30 to 0.35
Educational degree^12^	− 0.714	0.509	− 0.061	0.162	0.29 to 0.95
Occupation^13^	− 0.071	0.207	− 0.016	0.732	− 0.48 to 0.34
German language skills^14^	< 0.001	0.051	< 0.001	0.994	− 0.10 to 0.10
Sense of belonging to Germany^15^	− 0.166	0.109	− 0.082	0.129	− 0.38 to 0.05

Predictors of Generalized Anxiety Symptoms^2^					
Institutional verbal violence^6^	0.023	0.005	0.238	**< 0.001**	0.01 to 0.03
Migrant group^7^	− 0.181	0.185	− 0.046	0.330	− 0.55 to 0.18
Life satisfaction^8^	− 0.213	0.046	− 0.236	**< 0.001**	− 0.30 to − 0.12
Sense of belonging to the country of origin^9^	− 0.181	0.084	− 0.091	**0.032**	− 0.35 to − 0.02
Sex^10^	0.032	0.163	0.009	0.846	− 0.29 to 0.35
Age	− 0.007	0.008	− 0.043	0.365	− 0.02 to 0.01
Marital status^11^	− 0.107	0.166	− 0.028	0.519	− 0.44 to 0.22
Educational degree^12^	− 0.359	0.513	− 0.030	0.484	−1.37 to 0.65
Occupation^13^	0.258	0.209	0.055	0.217	− 0.15 to 0.67
German language skills^14^	− 0.093	0.051	− 0.082	0.071	− 0.19 to 0.01
Sense of belonging to Germany^15^	− 0.182	0.110	− 0.087	0.097	− 0.40 to 0.03

^1^PHQ-2: Patient Health Questionnaire—Depression Module, sum score, range: 0–6 (higher values are linked to higher symptom severity); ^2^GAD-2, Generalized Anxiety Disorder Scale, sum score, range: 0–6 (higher values are linked to higher symptom severity); ^3^B: regression coefficient; ^4^SE: standard error; ^5^CI: confidence interval; ^6^verbal violence in German public institutions, average sum score, range: 0–96 (higher values are linked to higher experience of institutional verbal violence); ^7^0: refugee; 1: voluntary migrant; ^8^life satisfaction, sum score, range: 0–10 (higher values indicate higher life satisfaction); ^9^sense of belonging to the country of origin, sum score, range: 0–3 (higher values are linked to higher sense of belonging); ^10^1: female; 2: male; ^11^0: single; 1: relationship; ^12^0: no; 1: yes; ^13^0: no; 1: yes; ^14^German language skills, sum score, range: 0–8 (higher values are linked to higher German language skills); ^15^sense of belonging to Germany, sum score, range: 0–3 (higher values are linked to higher sense of belonging); significant p values are marked in bold

#### Regression analysis with institutional verbal violence as a predictor variable

In the regression analysis with IVV as predictor variable and PHQ-2 as outcome variable, being a refugee (β = −0.12; *p* = 0.02), low life satisfaction (β = −0.26; *p* < 0.001) and a higher amount of experiencing IVV (β = 0.15; p 0.002) were significantly associated with increased symptoms of depression. The explained variance was 18%.

A low sense of belonging to the country of origin (β = −0.09; *p* = 0.03), low life satisfaction (β = −0.24; *p* < 0.001) and more frequent experiences of IVV (β = 0.24; *p* < 0.001) were significantly associated with increased symptoms of generalized anxiety. The explained variance was 22%.

#### Regression analysis with discrimination as a predictor variable

Perceived discrimination was significantly associated with increased symptoms of depression (β = 0.23; *p* < 0.001) and generalized anxiety symptoms (β = 0.26; *p* < 0.001) [see Additional file 2].

## Discussion

To our knowledge VIOLIN is the first project investigating migrants’ experiences of IVV in Germany and its association with their mental health states. Migrants experienced IVV most frequently in public transport, immigration offices, doctors’ offices, hospitals, and city council/district offices. Female voluntary migrants experienced IVV significantly more often than their male counterparts. There were no significant gender differences between female and male refugees in their experiences of IVV. Refugees showed significantly more depressive symptoms than voluntary migrants. Being a refugee, low life satisfaction and frequent experiences of IVV were significantly associated with increased depressive symptoms. Regarding generalized anxiety disorder, life satisfaction, experiences of IVV as well as a low sense of belonging to the country of origin were significant predictors for elevated symptom severity.

In terms of sociodemographic factors, migrants form a heterogeneous group. This makes comparisons within our sample difficult. The gender distribution in the group of refugees corresponds to that in Germany [30]. Yet, the proportion of women in the group of voluntary migrants is above average [31]. In addition, our sample has an above-average level of education [32]. These aspects must be considered in the following sections.

On average, migrants from our sample report to experience IVV rarely and discrimination less than once a year. In the institutional context, studies about the situation in Germany mostly examined migrants’ experiences of discrimination [14, 18], while no studies seem to exist on IVV. Since 2014, surveys have reported low levels of discrimination against migrants and refugees in Germany [21, 33]. However, people with a migration background experience discrimination significantly more often than other people [21, 33]. Furthermore, migrants’ experiences of discrimination in Germany seem to have increased in recent years [22, 34]. In a German study 35% of migrants reported having been discriminated against sometimes or frequently in the past year [22]. The German Centre for Integration and Migration Research examined in 2022 the experiences of discrimination of more than 21.000 people in Germany [4]. Using the EDS, around 90% of the visually recognizable people in the sample reported having experienced at least one of the forms of discrimination recorded in the scale in their everyday lives [4]. In general, awareness of the various forms of discrimination has also increased [34].

We could demonstrate that there are no significant differences in the experience of IVV and discrimination between refugees and voluntary migrants. In contrast to this, female migrants, particularly voluntary migrants, experience significantly more IVV compared to male migrants. This gender-specific difference was not found in the experiences of discrimination. This means that a larger group is affected by interactions perceived as problematic when VV is used as a measure. In contrast, the report of the National Discrimination and Racism Monitor 2023 by the German Center for Integration and Migration Research states that women experience less subtle discrimination on average than men [4]. However, the level of actual discrimination may differ from the level of perceived discrimination [35].

As for the institutions where IVV is mainly experienced, our results confirm the results of earlier studies on discrimination. The institutions where discrimination is most frequently perceived are agencies, authorities and the healthcare sector [4, 18, 23]. Compared to discrimination, including physical or sexual violence, IVV is more difficult to detect and establish retrospectively, as it results from the participants’ subjective interpretations and does no direct physical trace [1]. Moreover, in the context of migration, it can be masked by aspects such as language barriers and cultural differences [1]. The affected person may assume that the linguistic behavior perceived as problematic is simply due to intercultural differences in communicating. As a result, institutional representatives may have fewer inhibitions about being verbally rude with migrants. Our findings indicate that migrants experience IVV not on a daily base. However, mental distress may be caused by IVV in the reported institutions such as public transport, immigration authorities and health care.

We could demonstrate by our regression analyses that the experience of IVV is related to reduced mental health both of voluntary migrants and refugees. This finding is also consistent with previous studies that have reported discrimination to be negatively associated with mental health [7–11]. In multiple regression analyses, the effect of discrimination is slightly higher compared to the effect of the experience of IVV. One explanation might be that the EDS investigates everyday life [26], while the IVV questionnaire includes questions about specific situations in German institutions. By surveying a broader spectrum (not only institutions, not only verbal violence) it may be possible to capture stronger effects on mental health.

Manifestations of mental distress, depression and anxiety are significantly higher in our study population than in the German general population [36]. In line with our results, in the 2016 and 2017 IAB-BAMF SOEP survey of refugees in Germany, refugees revealed more depressive symptoms in PHQ-4 compared to the German population average [37]. As has been shown in numerous studies, prevalence scores for depression and anxiety among migrants vary by host country [14, 38], country of origin [39–41], migrant subgroup [42], and generation [40]. Displaced persons are more likely to exhibit psychological distress [14, 43], which was also confirmed in our survey. In our sample, the prevalence scores for refugees of 42% for depression and 34% for anxiety disorders are slightly higher than the prevalence scores for refugees in similar studies [44, 45]. Selection effects of the online study may have played a role here, so that those migrants and refugees affected with a high symptom burden were more likely to participate in the anonymous study, however the opposite could also be the case. Refugees are more likely to experience depression compared to voluntary migrants [14, 15]. In our sample, they exhibited significantly more depressive symptoms than voluntary migrants. Studies show that, compared to voluntary migrants, refugees are exposed to more pre-migratory stressors such as war and persecution, the flight itself, and post-migratory stressors such as administrative procedures in the context of obtaining residence permits which put a strain on their mental health [3]. The meta-analysis by Blackmore et al. [44] showed prevalence scores for refugees of 31.5% for depression and 11% for anxiety. For refugees exposed to an armed conflict, Mesa-Vieira et al. [45] reported in a meta-analysis prevalence rates of 25% for major depression and 14% for general anxiety disorders. The meta-analysis by Lindert et al. found prevalences for anxiety disorder between 6% and 44% for migrant workers and between 5% and 90% for refugees [46]. Regarding anxiety disorders the comparison between refugees and voluntary migrants in our sample was not significant, but a tendency was evident. With a mean of 2.09 in the PHQ-2 and of 2.05 in the GAD-2, our total sample revealed significantly more symptoms of depression and anxiety than the German general population with a mean of 0.97 in the PHQ-2 and of 0.83 in the GAD-2 [36]. In scientific literature, unfortunately only a few studies can be found that differentiate between refugees and voluntary migrants [47]. However, prevalence values are known to vary widely across studies, as migrants represent a very heterogeneous group [46, 48]. Yet, some studies didn´t find a difference between the mixed migrants population (voluntary migrants and refugees) and the general German population [49], especially not for voluntary migrants considering prevalences for depression and anxiety [50]. Certain factors such as cultural adjustment stress, discrimination in the host country, pre-migration trauma and displacement or uncertain immigration status [8] may contribute to an increased level of distress symptoms among migrants in our sample and could also explain the increased prevalence of depressive symptoms among refugees compared to voluntary migrants.

Gender was surprisingly not a significant predictor in our analysis. In literature, female migrants showed significantly higher depression [14, 16] and anxiety symptoms [17] compared to male migrants. The gender differences for depression [51] and generalized anxiety symptoms [52] are also evident in the general German population. It is also possible that gender is no longer significantly associated with poorer mental health in our study population when other socio-demographic characteristics such as education level are considered.

According to a meta-analysis by Brance et al. [53], social identification, both with the host country and with the home country community, protects ethnic minorities and migrants from depression and anxiety. The feeling of migrants of belonging to their country of origin, as a form of group affiliation, is also a negative significant predictor of anxiety disorders in our analysis (in the regression analysis with IVV as a predictor variable). One could assume that this feeling is a form of social identification, which acts as a protective factor against anxiety symptoms [53]. Social identification also correlates with other factors such as trauma and persecution [53], which in turn have a negative impact on mental health.

### Strengths and limitations

First of all, the strength of the study is the novelty of the construct of IVV, developed on the basis of interdisciplinary cooperation, the size of the sample and the recruitment through different channels such as social media (Facebook, Instagram), churches and public institutions, e.g. integration councils and anti-discrimination bodies. In addition, the questionnaire could be answered in nine different languages and equal access was ascertained for most ethnic groups. The questionnaire on IVV included 24 questions. This diversity may be considered as another strength as it increases the likelihood that different aspects of experiences with IVV were made measurable.

However, due to the cross-sectional design of the study, no causal conclusions can be drawn. But the link between symptoms of depression and anxiety and a higher perceived level of discrimination has already been proven [54]. One weakness was the size of the questionnaire which required a significant time effort to complete. Thus, some participants did not complete the survey. It is possible that migrants with poorer mental health did not complete the questionnaire and were therefore excluded from the analysis. The high proportion of participants with a high level of education and the high proportion of women in the group of voluntary migrants limit the representativeness of our sample. Recruitment also took place through university networks, which could be one reason why mainly young, well-educated migrants were reached. Education can serve as a resource to better integrate into society and exercise rights, which can potentially lead to less experience of IVV and better mental health. But integration into society also leads to increased sensitivity to perceived discrimination, which is known as the integration paradox [55]. In an average composition of the cohort, different values of perceived IVV are to be assumed.

Future research on the experience of IVV and its impact on mental health is needed to confirm recent findings and collect new results. For this, prospective studies with different ethnic groups need to be conducted so that the impact of IVV can be examined. In addition to the association with depression and generalized anxiety disorder, other disorders such as posttraumatic stress disorder should also be considered. In larger, more representative samples, gender differences must be looked at as well in order to verify the current results. In this context, it is also important to distinguish gender differences in the experience of IVV between female and male migrants as well as between women and men in the general population. Other obvious differences such as visible minorities could be included as well as the difference between the experience and perception of IVV.

## Conclusion

IVV is highly associated with the state of migrants’ mental health and circumstances in the host country contribute to this. This is of high importance as migrants, especially refugees, in Germany have a significantly higher risk of depressive and anxiety symptoms compared to the general population.

To gain further insight specifically on IVV, further studies should be conducted in individual institutions, focusing on gender differences. Special attention should be paid to immigration offices, health care institutions and city councils/district offices.

To reduce IVV in the future and to raise awareness and sensitize on the issue, courses could be set up training employees in these institutions about where discrimination begins and about its effects on the mental health of migrants. Also, guidance is needed on how to intervene (and report) if other people at their workplace discriminate against migrants. Training and monitoring of the effectiveness of the training would allow to directly improve communication with voluntary migrants and refugees in institutions.

To minimize IVV in public places, for example in public transport, the general population could be reached directly, i.e. through advertising campaigns. Society benefits from such measures, but perhaps there is not yet sufficient awareness for this.

## Supplementary Information

Below is the link to the electronic supplementary material.

Supplementary Material 1

Supplementary Material 2

## Data Availability

The dataset analyzed during the current study is available from the corresponding author on reasonable request.

## Abbreviations

IVV: Institutional verbal violence
VV: Verbal violence
VIOLIN: Verbal Violence against Migrants in Institutions
