# Supplementary material for: Results from the VIOLIN study: verbal violence against voluntary migrants and refugees in German public institutions, discrimination and their association with mental health—an online-cross-sectional study: Institutional verbal violence and discrimination against migrants and their impact on mental health
Source: BMC Public Health. 2025 Aug 27;25:2953. doi: 10.1186/s12889-025-24363-y (PMC12382206; doi:10.1186/s12889-025-24363-y)
Supplement: Supplementary file 1 — Supplementary Material 1 [file 12889_2025_24363_MOESM1_ESM.docx]

Additional file 1. Mean values of the nine most frequent countries of origin for PHQ-4, PHQ-2, GAD-2, institutional verbal violence and EDS.

| Country of Origin | N | PHQ-4^1^  M (SD) | PHQ-2^2^  M (SD) | GAD-2^3^  M (SD) | Institutional Verbal violence^4^  M (SD) | EDS^5^  M (SD) |
| --- | --- | --- | --- | --- | --- | --- |
| Afghanistan | 53 | 4.28 (3.24) | 2.08 (1.69) | 2.10 (1.76) | 18.98 (15.88) | 10.49 (10.00) |
| Argentina | 20 | 3.15 (2.98) | 1.55 (1.32) | 1.60 (1.85) | 27.40 (19.63) | 10.45 (9.87) |
| Ecuador | 25 | 3.72 (3.62) | 1.88 (1.81) | 1.84 (1.86) | 25.00 (20.20) | 11.08 (9.81) |
| Germany | 61 | 3.85 (2.87) | 1.98 (1.48) | 1.87 (1.61) | 22.49 (17.59) | 12.20 (9.62) |
| Iran | 26 | 4.38 (3.28) | 2.19 (1.90) | 2.19 (1.67) | 30.42 (18.45) | 13.73 (9.14) |
| Mexico | 41 | 3.51 (3.44) | 1.63 (1.70) | 1.88 (1.96) | 29.80 (17.76) | 12.73 (9.03) |
| Poland | 53 | 3.94 (3.10) | 2.08 (1.71) | 1.87 (1.51) | 15.77 (13.83) | 6.58 (7.21) |
| Syria | 48 | 5.17 (3.52) | 2.63 (1.79) | 2.54 (1.92) | 29.10 (18.45) | 13.15 (9.63) |
| Turkey | 29 | 3.59 (2.85) | 2.03 (1.52) | 1.55 (1.55) | 19.14 (14.75) | 8.83 (6.47) |

*^1^ PHQ-4: Patient Health Questionnaire, sum score, range: 0–12 (higher values are linked to higher psychological distress); 2 PHQ-2: Patient Health Questionnaire—Depression Module, sum score, range: 0–6 (higher values are linked to higher psychological distress); 3 GAD-2, Generalized Anxiety Disorder Scale, sum score, range: 0–6 (higher values are linked to higher psychological distress); 4 verbal violence in German public institutions, average sum score, range: 0–96, (higher values are linked to higher experience of institutional verbal violence); 5 EDS: Everyday Discrimination Scale, average sum score, range: 0–50 (higher values are linked to higher levels of perceived discrimination).*
