# Supplementary material for: Results from the VIOLIN study: verbal violence against voluntary migrants and refugees in German public institutions, discrimination and their association with mental health—an online-cross-sectional study: Institutional verbal violence and discrimination against migrants and their impact on mental health
Source: BMC Public Health. 2025 Aug 27;25:2953. doi: 10.1186/s12889-025-24363-y (PMC12382206; doi:10.1186/s12889-025-24363-y)
Supplement: Supplementary file 2 — Supplementary Material 2 [file 12889_2025_24363_MOESM2_ESM.docx]

Additional file 2. Multiple linear regression analyses including perceived discrimination and socio-demographic and migration-related characteristics as predictor variables.

| Predictors of Depression Symptoms^1^ | B^3^ | SE^4^ | β | p | 95% CI^5^ |  |
| --- | --- | --- | --- | --- | --- | --- |
| EDS^6^ | .040 | .008 | .226 | **<.001** | .02 to .06 |  |
| Migrant group^7^ | -.444 | .182 | -.117 | **.015** | -.80 to -.09 |  |
| Life satisfaction^8^ | -.210 | .045 | -.242 | **<.001** | -.30 to -.12 |  |
| Sex^9^ | .067 | .159 | .019 | .672 | -.24 to .38 |  |
| Age | -.004 | .007 | -.025 | .598 | -.02 to .01 |  |
| Marital status^10^ | .061 | .162 | .017 | .709 | -.26 to .38 |  |
| Educational degree^11^ | -.811 | .503 | -.069 | .108 | -1.80 to .18 |  |
| Occupation^12^ | -.061 | .205 | -.014 | .766 | -.47 to .34 |  |
| German language skills^13^ | -.022 | .050 | -.020 | .659 | -.12 to .08 |  |
| Sense of belonging to the country of origin^14^ | -.103 | .083 | -.054 | .213 | -.27 to .06 |  |
| Sense of belonging to Germany^15^ | -.104 | .109 | -.052 | .341 | -.32 to .11 |  |
| Predictors of Generalized Anxiety Symptoms^2^ |  |  |  |  |  |  |
| EDS^6^ | .047 | .009 | .255 | **<.001** | .03 to .06 |  |
| Migrant group^7^ | -.195 | .185 | -.049 | .293 | -.56 to .17 |  |
| Life satisfaction^8^ | | -.206 | .046 | -.229 | **<.001** | -.30 to -.12 |
| Sex^9^ | -.045 | .161 | -.012 | .780 | -.36 to .27 |  |
| Age | -.007 | .008 | -.043 | .363 | -.02 to .01 |  |
| Marital status^10^ | -.051 | .165 | -.013 | .760 | -.37 to .27 |  |
| Educational degree^11^ | -.477 | .512 | -.039 | .352 | -1.48 to .53 |  |
| Occupation^12^ | .282 | .208 | .060 | .176 | -.13 to .69 |  |
| German language skills^13^ | -.117 | .051 | -.103 | .023 | -.22 to .02 |  |
| Sense of belonging to the country of origin^14^ | -.160 | .084 | -.080 | .059 | -.33 to .01 |  |
| Sense of belonging to Germany^15^ | -.141 | .111 | -.067 | .206 | -.36 to .08 |  |

*^1^ PHQ-2: Patient Health Questionnaire—Depression Module, sum score, range: 0–6 (higher values are linked to higher symptom severity); ^2^ GAD-2, Generalized Anxiety Disorder Scale, sum score, range: 0–6 (higher values are linked to higher symptom severity); ^3^ B: regression coefficient; ^4^ SE: standard error; ^5^ CI: confidence interval ;^6^ EDS: Everyday Discrimination Scale, average sum score, range: 0–50, (higher values are linked to higher levels of perceived discrimination); ^7^ 0: refugee; 1: voluntary migrant; ^8^ life satisfaction, sum score, range: 0-10 (higher values indicate higher life satisfaction); ^9^ 1: female; 2: male; ^10^ 0: single; 1: relationship; ^11^ 0: no; 1: yes; ^12^ 0: no; 1: yes; ^13^ German language skills, sum score, range: 0-8 (higher values are linked to higher German language skills); ^14^ sense of belonging to the country of origin, sum score, range: 0-3 (higher values are linked to higher sense of belonging); ^15^ sense of belonging to Germany, sum score, range: 0-3 (higher values are linked to higher sense of belonging).*
